# 

**Published:** 1851-03

**Authors:** 


### ORIGINAL COMMUNICATIONS.

### ESSAYS AND CASES.

ART. I.—Clininal Observations in Private Practice. By Dr. E. B. HASKINS, of Clarksville, Tenn. - - - - 185
ART. II.—Translations from Achives Genrales de Medicine, for December, 1850. Translated by WM. H. COBB, M. D., of Louisville, Ky. - - - - - - 191

### SELECTIONS FROM FOREIGN AND HOME JOURNALS.

On the Exhibition of Quinine in the Febrile Paroxysm. - 198
Treatment of Strumous Disorders by means of Preparation of Leaves of Walnut, - - - - - - - - 204
Traumatic Tetanus cured by Frictions with Tincture of Belladonna, - - - - - - - - - - - 207
Hare-Lip in France, - - - - - - - - 208
On the comparative liability of Males and Females to insanity, and their comparative curability when Insane. - - 209
Sick Headache cured by Full Inspirations, - - - - 210
Errors in Diagnosis. - - - - - - - - - - - 211
Acephalous Monster, - - - - - - - - - - 212
On the comparative value of the Diagnostic Signs of Pneumonia, - - - - - - - - - - - - - 214
Medicated Soaps for the Treatment of Skin Disease, - 217
Differential Diagnosis of Gastralgia and other more Serious Affections of the Stomach, - - - - - - - 217
Diagnostic Value of Epigastric Pains, - - - - - - 219

On Morbid Poisons, - - - - - - - - 220
Treatment of Habitual Constipation, - - - - - - 228
Uses of the Nitro-Muriatic Acid Bath, - - - - - 230
On the Symptomatological Value of Palpitation, - - - 232
Dislocation of Femur reduced on sixty-eighth day, by Jarvis's Surgical Adjuster, - - - - - - - 235
The Kousso—A New Anthelmintic, - - - - - - 236
Clinical Lecture on the Kousso, - - - - - - 242
Savine in the Treatment of Habitual Abortion, - - - 246
Treatment of Puerperal Fever, - - - - - - 247
A Japanese Remedy for Sterility, - - - - - - - 255
Indian Hemp as an Oxytoxic, - - - - - - - 256

REVIEWS.

Art. I.—History of the Strange Sounds or Rappings, heard in Rochester and Western New York, and usually called the Mysterious Noises, &c. By D. M. Dewey, Rochester, 1850.
Exposition of the Rochester Knockings. By Austin Flint, M. D., Charles Lee, M. D., and C. B. Coventry, M. D. 257
Art. II.—United States Exploring Expedition, during the years 1838, 1839, 1840, 1841, 1842, under the command of Charles Wilkes, U. S. N. Vol. ix.
The Races of Man, and their Geographical Distribution. By Chas. Pickering, M. D., member of the scientific Corps attached to the Expedition.
The Races of Men: A Fragment. By Robert Knox, M. D., Lecturer on Anatomy, and corresponding member of the National Academy of Medicine of France.
The New Englander—1850.
The North American Review—1850.
The Doctrine of the Unity of the Human Race examined on the principles of Science. By John Bachman, M. D. Professor of Natural History, College of Charleston, S. C. 265

ORIGINAL INTELLIGENCE.

Demonstrative Midwifery, - - - - - 275

